# Epidemiology and hospitalization costs of chronic kidney disease in Romania

**DOI:** 10.1186/s13561-025-00614-x

**Published:** 2025-04-25

**Authors:** Ildiko Aliz Bradacs, László-István Bába, László Lorenzovici, Andreea Mihaela Precup, Szabolcs Farkas- Ráduly, Gyongyi Tar, Vasile Nastase, Lucia Georgeta Daina, Raul Bozu, Gyula Jozsef Nagy, Dimitrie Cristian Siriopol, Dorel Sandesc, Ovidiu Horea Bedreag, Florin Buicu, Gabriel Mircescu, Gener Ismail

**Affiliations:** 1 Doctoral School of Biomedical Sciences, Faculty of Medicine and Pharmacy, Oradea, 410087 Romania; 2 Dr. Mircea Pop City Hospital, Marghita, Romania; 3 Syreon Research Romania, Tirgu Mures, Romania; 4 George Emil Palade University of Medicine, Pharmacy, Science, and Technology of Targu Mures, 540142 Targu Mures, Romania; 5 Sapientia Hungarian University of Transilvania, Targu Mures, Romania; 6 Fundeni Clinical Institute, Bucharest, Romania; 7 Department of Psycho-Neurosciences and Recovery, Faculty of Medicine and Pharmacy, University of Oradea, Oradea, Romania; 8 Clinical County Emergency Hospital of Oradea, Oradea, Romania; 9 Doctoral School, Faculty of Health Sciences, University of Pecs, Pecs, Hungary; 10 Ștefan cel Mare, University of Suceava, Suceava, Romania; 11 County Emergency Hospital Suceava, Suceava, Romania; 12 Anaesthesia and Intensive Care Medicine Department, Victor Babes University of Medicine and Pharmacy, Timisoara, Romania; 13 Department of Nephrology, “Carol Davila” University of Medicine and Pharmacy, 050474 Bucharest, Romania; 14 Department of Nephrology, “Dr. Carol Davila” Teaching Hospital of Nephrology, Bucharest, Romania

**Keywords:** Chronic kidney disease, Dialysis, Hospitalization cost, Budget impact

## Abstract

**Background:**

Chronic kidney disease or chronic renal failure is a progressive condition defined as abnormalities of kidney structure or function, present for longer than 3 months. It is estimated to affect more than 10% of the general population worldwide. Management of CKD represents an especially large burden for the health systems of low- and middle-income countries, and it has been recognized as a leading public health problem. Previous research articles reported an age-adjusted prevalence of 7.6% for Romania, but the hospital costs generated by CKD are unknown. The present research article aimed to measure the hospital costs and one-year national healthcare budget impact of CKD, excepting the chronic care costs of RRTs.

**Methods:**

In this retrospective study we reviewed the electronic health records of 4 University, 3 County and 5 City hospitals from 1^st^ of January 2019 to 31^st^ of December 2019 in order to calculate costs related to hospitalization due to chronic kidney disease. Inclusion criteria were defined as: CKD-related diagnostic codes or dialysis-related procedures in medical cases (without surgical interventions). KDIGO severity grades 1-5 were considered, including dialysis costs. The costs generated by the chronic care of RRTs were not considered here. Hospitalization cost calculation was based on hospital controlling methodology including direct, indirect and overhead costs. For the national-level burden study, we analyzed the health claim records of all public and private hospitals for 2019.

**Results:**

In 2019 a total number of 229 276 cases reported chronic kidney disease in Romania. The average hospital costs per patient episode was €917.1, with significantly higher costs in cases with complications or higher severity grades. The total hospitalization cost-related budget impact in 2019 was €210 million.

**Conclusions:**

The high hospitalization costs of CKD (representing 2.6% of the NHIH budget, not considering the funds for sick leave) cause major impact on the national health payer`s budget. Preventive strategies, early diagnosis and management as well as health education measures could act as means of mitigation. Our results should warn the public health policy decision makers about the importance of this disease.

**Supplementary Information:**

The online version contains supplementary material available at 10.1186/s13561-025-00614-x.

## Background

According to the Kidney Disease Improving Global Outcomes (KDIGO 2012) definition, chronic kidney disease (CKD) is defined as abnormalities of kidney structure or function, present for longer than 3 months, with implications for health [1–3]. CKD is a chronic and progressive condition that represents a serious concern for the healthcare systems, because of several reasons.

Most importantly, according to a recent estimation, it affects about 10% of the general population worldwide, amounting to more than 800 million individuals [1, 4].

This disease is causing considerable suffering to the patients. A recent meta-analysis conducted by C. Freeman and collab. revealed a significantly lower quality of life (QoL) for CKD patients [5]. More than that, more severe stages of the disease were associated with reduced QoL [5].

CKD is of great concern not only from a humanistic point of view, but also from the economic burden`s point of view. Its complications and comorbidities are associated with high costs. These have risen considerably after the mid-20^th^ century, when renal replacement therapies (RRTs) became available, making possible the long-term application of lifesaving but costly treatment for patients with end-stage kidney disease (ESKD) [6]. According to a recent research article published as part of the inside CKD research program, the mean annual costs (calculated for 31 states worldwide) associated with hemodialysis were $57 334, those for peritoneal dialysis and incident renal transplant were $49 490 and $75 326, respectively [4]. Epidemiological projections are sober, since the number of patients receiving RRTs is projected to double by 2030 [7]. Not only do the aforementioned RRTs account for the high burden of the disease, but also the higher, all-cause hospitalization rate, especially in the older adults. In this regard, Wong and collab. demonstrated a dramatic increase in all-cause hospitalization rate in large cohort of CKD patients in *„very high risk”* patients compared to inferior risk categories according to the KDIGO classification [8]. Later published conference papers also confirmed this observation [9]. Similar results were published regarding ten of the most common specific hospitalization causes by the team led by Iwagami [10].

Additionally, there are subpopulations with higher risk for developing CKD, such as women, older individuals, racial minorities, and patients with certain comorbidities (diabetes mellitus, hypertension, obesity) [1, 11, 12]. Because of the increase in prevalence of the mentioned risk factors worldwide, the incidence of CKD is growing considerably [11].

Considering the higher prevalence of the mentioned risk factors in Eastern Europe compared to Western Europe, the number of CKD cases is expected to be higher in Eastern Europe. Accordingly, recent evidence has confirmed this hypothesis [13]. Earlier publications reported an age-standardized prevalence of 7.2-7.6 % in Romania [14, 15]. Cost data in the literature are scarce, but a previous article reported per patient CKD management costs of €614.2, €740.3, €818.2, and €1237.6 for severity stages G3a, G3b, G4 and G5, respectively [4]. Although there has been data published about the epidemiology and one-year treatment cost of CKD in Romania, the hospital costs of this disease, stratified by severity grade have not been published so far.

In this retrospective study, we aimed to measure the hospitalization costs and the total budget impact of CKD cases in Romania in 2019.

## Methods

This retrospective study comprised two analyses. In the first analysis, we sought for hospital costs of CKD cases. The aim of the second analysis was to estimate the total number of cases nationally, which was a prerequisite for the calculation of the national level hospital cost for 2019. Surgical and other types of interventions, emergencies and cases that generated cost mainly because of other health issues were excluded (detailed in Table 1). Since CKD is a condition that correlates with higher incidence of drug adverse reactions, longer LoS andfrequent ICU admissions (especially if drugs excreted in the kidneys are administered) even in patients that underwent surgical interventions, we consider the cost measurement of the present study to be conservative.

**Table 1:** Inclusion and exclusion criteria of CKD cases

**Inclusion criteria**	**Exclusion criteria**
A. Cases that reported CKD-related diagnosis codes (primary and secondary) as specified in Supplementary Table 1 B. Dialysis-related procedures codes as specified in Supplementary Table 2 C. Case type: medical cases.	1. Kidney transplant 2. Renal failure codes other than those specified in Supplementary Table 1 (e.g. Acute renal failure) 3. Surgical and other type of cases except those that had kidney-related procedure codes 4. Cases that had surgical procedures for other organs/systems mentioned. 5. Acute emergencies (myocardial infarction, acute thrombosis/or bleeding, acute infections or reactivated chronic infections) 6. Acts of violence, trauma, poisoning

### Data sources, inclusion and exclusion criteria

For the extraction of epidemiological and financial data, electronic healthcare records were reviewed from two different sources. For the cost analysis, the financial data from twelve public hospitals were used, that had controlling system implemented, as detailed in Section "Results" (cost analysis). For the epidemiological analysis, we searched the claims database of all public and private hospitals, reported to the National Health Insurance House in 2019 [16].

A conservative approach is utilized in the present work. We included cases that had primary and secondary CKD-related diagnostic codes. The inclusion and exclusion criteria are shown in Table 1. Inclusion algorithm was: (A OR B) AND C. Medical cases were defined as cases without surgical procedure codes. Surgical cases were defined as cases having at least one surgical procedure code. The same criteria were applied for both the cost and the epidemiological analysis.

Neither patient-informed consent nor ethics committee approval were required, as anonymized data were used exclusively throughout the research. Detailed hospitalization costs, demographic and medical data (i.e., primary and secondary diagnoses) were extracted for each clinical case. Subsequent admissions were considered as separate patient episodes (PE).

### Cost analysis and complications

In the second analysis, we reviewed in detail the electronic health records from the reimbursement claims of twelve public hospitals from 1^st^ of January 2019 to 31^st^ of December 2019. In this analysis we aimed to measure the hospital costs of CKD, stratified by hospital level: our sample of twelve hospitals included 4 university, 3 county and 5 city hospitals. These were chosen from different geographical regions of Romania. All of these hospitals had cost controlling systems implemented and provided good quality costing data.

For cost measurement, a *„bottom-up”* retrospective approach was used in a conservative manner. Raw financial data extracted in national currency and were converted using an exchange rate of 4.7452 RON per 1 € [17]. Hospitalization cost calculation was based on controlling methodology, the types of costs considered are listed in Table 2.

**Table 2:** Types of hospital costs extracted

**Type of cost**	**Costs considered**
Direct costs	Medical and non-medical personnel salaries Drugs Healthcare and other materials
Internal services	Operating room costs Intensive care costs Laboratory Imaging Laundry Sterilization Catering
Administrative services	Purchased services Amortization Administrative services

Hospital costs by CKD stage were calculated in a subgroup analysis. Where available, CKD classification was extracted from the electronic healthcare records. For CKD staging, the 2012 KDIGO CKD guideline was used, based on glomerular filtration rate, as recommended by the National Kidney Foundation [3]. Estimated glomerular filtration rates (eGFR) were calculated from subsequent serum creatinine values using the 2009 CKD-EPI Creatinine Equation. Kidney damage with a duration of >3 months was considered CKD.

To study the impact of complications on the length of stay and costs of cases, we undertook a sub-stratification of the cases from the twelve sample hospitals based on the complications that occurred. The complications that we considered in this analysis were those that have pathological significance and occurred in at least 0.002% of cases (at least 50 cases in the sample). These were the following: hyperkalemia, volume depletion, major hypoglycemic events, fracture, anemia and heart failure.

### Epidemiological analysis and national level hospitalization costs

For the epidemiological and the national-level cost analysis we used the annual inpatient claims database of the National School of Public Health, Management and Professional Development, Bucharest to determine the total number of discharged CKD cases in 2019. The same inclusion and exclusion criteria were applied to those specified in the cost analysis (Table 1.). National-level hospitalization costs were calculated based on the number of cases treated at each hospital level and the mean costs per case, calculated for the corresponding hospital level. Prevalence calculations were based on the residential population of Romania that which was 19.4 million at 1^st^ of January 2019.

### Statistical analysis

For the comparison of the overall hospitalization costs and the drug costs between the cases of different severity grades and complications Kruskal-Wallis test with Dunn`s multiple comparison post-test was used with a statistical significance level of α=0.05. For the analysis, Garphpad Prism 5.0 and R version 4.4.2 (RStudio version. 2024.12.0 build 467) were used. For the analysis of the association between age and the severity grade of CKD, Fisher`s Exact Test with Monte Carlo simulation for p-values (10 000 simulations) was used. Comparison of the demographical hospitalization parameters of nationally admitted cases between males and females, the Mann-Whitney test was used.

## Results

### Inpatient costs of CKD

In our sample of twelve public hospitals, a total of 18 948 cases were reported with CKD diagnosis in 2019. This represents 8.3% of all cases nationally with CKD. Hospitalization cost analysis has been stratified according to hospital types as shown in Table 3. The highest costs have been reported in county hospitals (€1 032.1 and 670.5 mean and median, respectively, Table 3).

**Table 3:** Basic health care utilization and costing data of the CKD cases from the twelve sample hospitals enrolled in the cost-measurement study

**Hospital type**	**No of discharged cases from the sample hospitals (% of cases nationally)**	**Mean LoS ± SD**	**Median LoS (IQR)**	**Total mean cost/case (±SD) (€)**	**Total median cost per case (IQR) (€)**
University hospital	11 442 (10.4)	6.2±6.1	5 (2 - 8)	900.8±1660.3	545.1 (375.3-856.5)
County hospital	4 728 (6.3)	7.8±5.2	7 (4 - 10)	1032.1±1594.9	670.5 (460.5-1009.9)
City hospital	2 778 (6.4)	7.6±4.6	7 (5 - 9)	760.1±1101.0	556.6 (408.5-758.6)
**Total (sample hospitals)**	**18 948 (8.3)**	**6.9±5.7**	**6 (3 - 9)**	**912.9±1576.0**	**574.9 (391.4-880.9)**

*LoS* length of stay, *IQR* interquartile range, *SD* standard deviation

**Fig 1:**
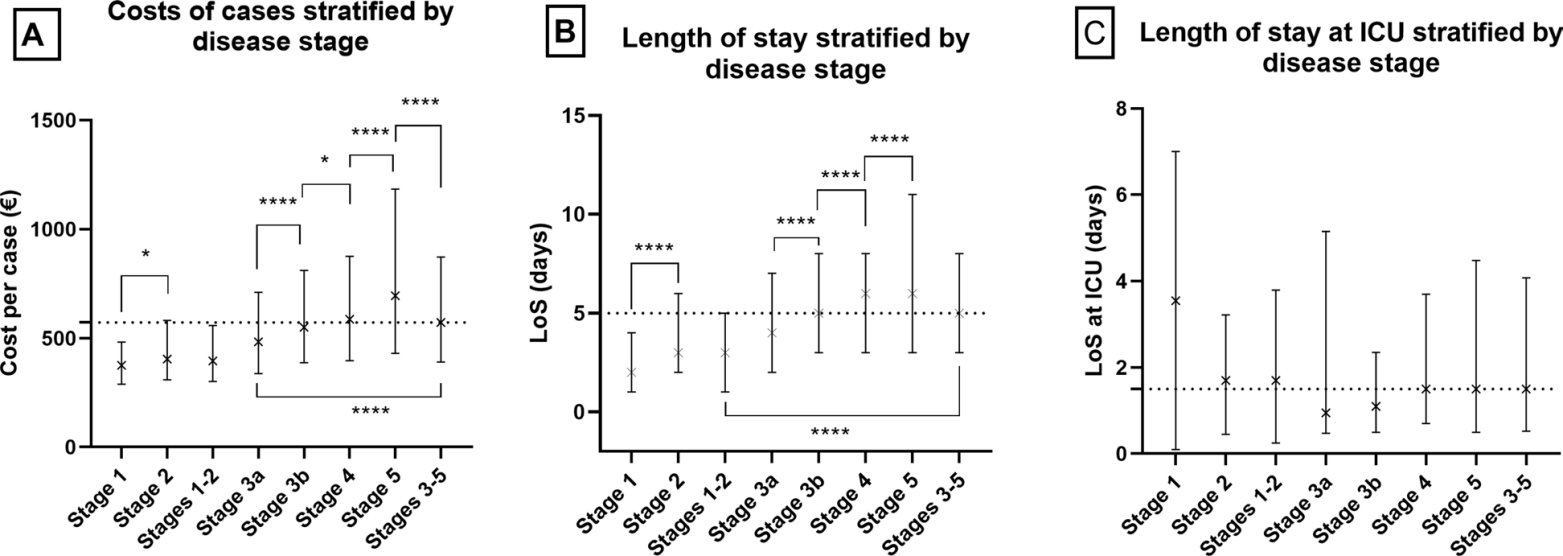
The impact of disease stage on costs and length of stay. **A** The hospitalization cost of CKD stratified by disease severity grades. **B** Length of stay stratified by disease severity grades. **C.** Length of stay at ICU stratified by severity grades. Medians and interquartile ranges are shown with statistically significant differences noted with * (*p*<0.05) and **** (*p*<0.0001) respectively. Medians of all studied cases are noted with dotted lines

Data regarding the disease severity grade were available for 9 408 out of 18 948 CKD cases discharged from the twelve sample hospitals, representing 49.7% of all cases. Costs increased with disease severity, with means ranging from €432.7 to €1147.2 corresponding to Stages 1 and 5 respectively, as shown in Table 4. LoS as well as LoS at ICU (intensive care unit) showed similar increasing trends as the costs did. Statistical analysis revealed a highly significant difference between the costs of cases of different severity grades (Kruskal Wallis test with Dunn`s multiple comparisons all pairs *p*<0.0001 except for Stage 1 vs. Stage 2 and Stage 3b vs. Stage 4 where *p*<0.5; Fig 1A). Similarly, LoS stratified by the severity grades showed statistically highly significant differences (*p*<0.0001; Fig. 1B). LoS at ICU showed no statistical differences stratified by disease severity (Fig. 1C).

**Table 4:** Number of CKD cases from the sample hospitals stratified by age groups and stages

	**Number of cases (% of all cases**^**a**^**)**	**<18**	**19-29**	**30-39**	**40-49**	**50-59**	**60-69**	**>70**
Nr. of cases Gr1	403 (4.3)	0	82 (0.9)	116 (1.2)	46 (0.5)	73 (0.8)	73 (0.8)	13 (0.1)
Nr. of cases Gr2	1 468 (15.6)	3	51 (0.5)	80 (0.9)	185 (2)	326 (3.5)	452 (4.8)	371 (3.9)
**Nr. of cases Gr1-2**	**1 871 (19.9)**	**3**	**133 (1.4)**	**196 (2.1)**	**231 (2.5)**	**399 (4.2)**	**525 (5.6)**	**384 (4.1)**
Nr. of cases Gr3a	1 511 (16.1)	3	25 (0.3)	46 (0.5)	106 (1.1)	189 (2.0)	467 (5.0)	675 (7.2)
Nr. of cases Gr3b	2 188 (23.3)	1	22 (0.2)	49 (0.5)	128 (1.4)	198 (2.1)	531 (5.6)	1 259 (13.4)
Nr. of cases Gr4	2 066 (22)	1	19 (0.2)	71 (0.8)	140 (1.5)	197 (2.1)	546 (5.8)	1 092 (11.6)
Nr. of cases Gr5	1 772 (18.8)	2	43 (0.5)	95 (1.0)	191 (2.0)	280 (3.0)	529 (5.6)	632 (6.7)
**Nr of cases Gr 3a-5**	**7 537 (80.1)**	**7**	**109 (1.2)**	**261 (2.8)**	**565 (6.0)**	**864 (9.2)**	**2 073 (22)**	**3 658 (38.9)**
**Nr. of cases Total (%)**	**9 408 (100.0)**	**10**	**242 (2.6)**	**457 (4.9)**	**796 (8.5)**	**1 263 (13.4)**	**2598 (27.6)**	**4 042 (43.0)**

^a^Calculated from the number of cases where KDIGO classification was available. Percentages lower than 0.1% are not presented

Since CKD is a condition that has an incidence that increases with age, we sought to analyze the impact of the age of the patients on the disease severity in our sample of twelve hospitals. When analyzing the association between the age and CKD stages, a highly significant association was found (age group x stage with a *p*-value of *p*<0.0001, Tab. 4), with a greater number of cases in older patients.

The cost of PE increased with the severity grade and ranged between (€ 432.6±262.1 and € 1 147.2±1 953.2 Table 5 and Fig. 1A).The LoS showed a similar pattern (Table 5). The costs induced by drugs showed a similar increase with the severity grades and ranged from € 16.26±37.64 to € 116.4±392.0 (Supplementary Fig. 1).

**Table 5:** Costs stratified by disease stage of the cases reported from the sample hospitals

**Disease severity stage**	**No of cases**	**% of sample**	**Mean cost per case ± SD (€)**	**Median cost per case (IQR) (€)**	**% of total costs**^**a**^	**LoS Mean±SD (days)**	**Median LoS (IQR)**	**Mean LoS at ICU**^**b**^	**Median LoS at ICU (IQR)**^**b**^	**Mean number of main complicatios±SD**
Stage 1	403	4.3	432.6±262.1	376.0 (291.8 – 2 289.3)	2.5	2.9±2.6	2 (1 - 4)	3.6±4.9	3.6 (0.1 -7.0)	0.3±0.5
Stage 2	1 468	15.6	482.2±295.1	404.6 (309.9 – 2 763.2)	10.3	3.9±3.0	3 (2 - 6)	2.0±1.7	1.7 (0.5 – 3.2)	0.4±0.5
**Total stages 1-2**	**1 871**	**19.9**	**471.5±289.0**	**396.0 (302.6 – 2 653.5)**	**12.8**	3.7±3.0	3 (1 - 5)	2.3±2.3	1.7 (0.3 – 3.8)	0.3±0.5
Stage 3a	1 511	16.0	594.4±424.2	484.0 (338.0 – 3 375.6)	13.1	5.0±3.7	4 (2 - 7)	2.6±2.7	1.0 (0.5 – 5.2)	0.6±0.6
Stage 3b	2 188	23.3	686.3±664.2	551.5 (387.9 – 3 845.1)	21.8	5.8±4.3	5 (3 - 8)	2.9±5.0	1.1 (0.5 – 2.35)	0.7±0.7
Stage 4	2 066	22.0	758.4±696.5	588.6 (397.0 – 4 151.2)	22.8	6.2±4.6	6 (3 - 8)	2.8±3.1	1.5 (0.7 – 3.7)	0.7±0.9
Stage 5	1 772	18.8	1 147.2±1 953.2	695.97 (431.0 – 5 605.2)	29.5	7.8±6.8	6 (3 - 11)	4.1±8.1	1.5 (0.5 – 4.5)	1.0±0.8
**Total stages 3a-5**	**7 537**	**80.1**	**796.0±1 231.9**	**572.89 (391.4 – 4 137.1)**	**87.2**	6.2±5.1	5 (3 - 8)	3.5±6.6	1.5 (0.5 – 4.)	0.8±0.7
**Total (sample hospitals)**	**9 408**	**100.00**	**731.5±1 576.0**	**533.2 (369.7 – 3 844.7)**	**100**	**5.7±4.8**	**5 (2 - 8)**	3.5±6.6	1.5 (0.5 – 4.0)	0.7±0.7

*LoS* length of stay, *IQR* interquartile range, *SD* standard deviation

^a^Calculated considering the total cost of cases that had severity grades specified

^b^Calculated from the cases that were admitted to ICU

**Fig. 2:**
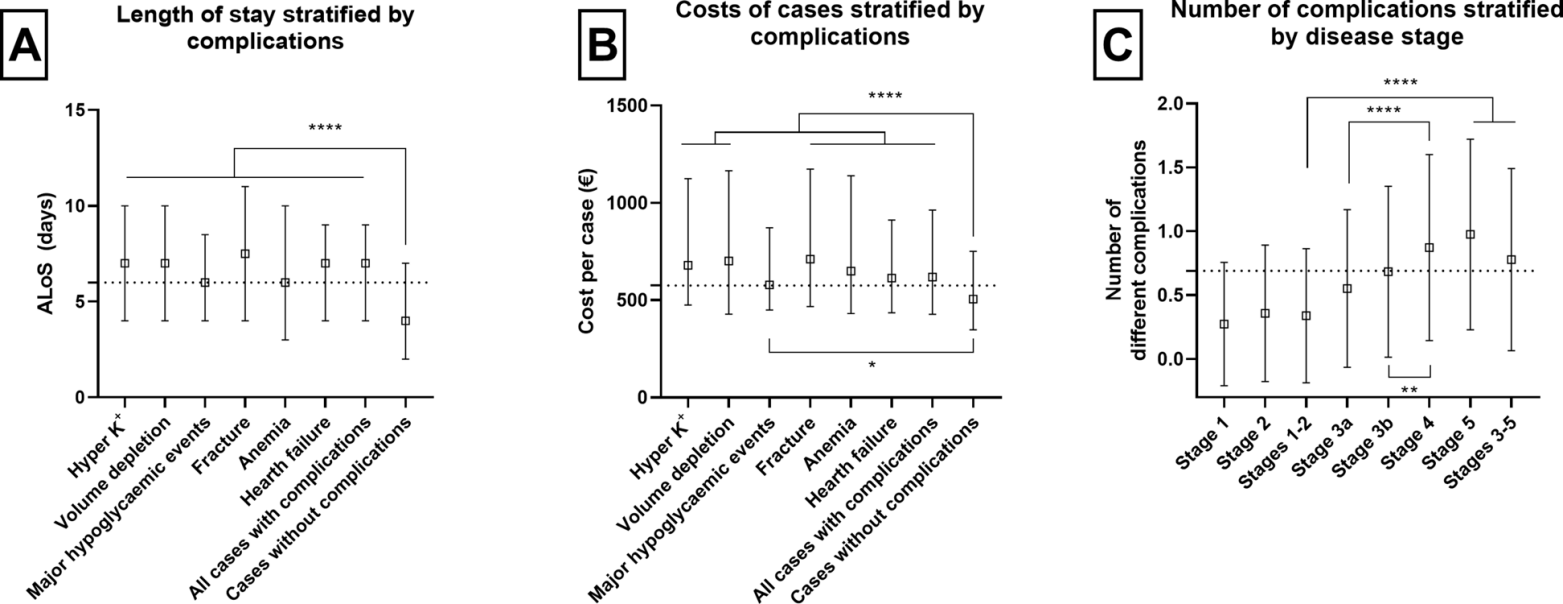
The importance of the complications of CKD cases. **A** The impact of complications on the length of stay. **B** The cost of cases stratified by complications. **C** The average number of complications. Medians and interquartile ranges are shown for length of stay and costs and means with standard deviation for number of complications. Statistically significant differences are noted with * (*p*<0.05) and **** (*p*<0.0001) respectively. Medians (A - length of case and B - costs) and mean (C – number of complications) of all studied cases appear with dotted lines

The cost of complications had an important impact on the cases and resulted significant differences in terms of LoS and costs (Table 6, figure 2A-B). The most frequently encountered complication that correlated with kidney disease was heart failure, followed by anemia while the prevalence of fractures and hypoglycemic events in our sample was low (Table 6). The costs were considerably higher in patients that suffered fractions.

**Table 6:** The main complications of CKD cases and corresponding costs in the twelve sample hospitals

**Complications of CKD cases**	**Number of cases from sample hospitals**	**Mean LoS ± SD (days)**	**Median LoS (IQR)**	**Mean cost per case ± SD (€)**	**Median cost per case (IQR)**	**Mean number of main complications ± SD**^**a**^
Hyper K^+^	1 581	7.7±5.8	7 (4.0-10.0)	1 150.2±2 048.8	679 (1 125.5 – 1 124.7)	1.9±0.7
Volume depletion	568	7.8±6.0	7 (4.0-10.0)	1 202.5±332.1	701.8 (1 016 – 1 164.8)	1.9±0.8
Major hypoglycemic events	153	7.0±5.1	6 (4.0-8.50)	784.8±757	578.7 (1 066 - 871.2)	1.8±0.7
Fracture	66	9.0±8.5	7.5 (4.0-11.0)	1 524.5±371.1	710.4 (1 109 – 1 173.4)	1.9±0.7
Anemia	4 898	7.8±6.7	6.0 (3.0-10.0)	1 109.1±1 897.3	649.3 (1 024 – 1 139.9)	1.5±0.6
Hearth failure	7 496	7.5±5.2	7 (4.0-9.0)	939.9±1 489.7	613.5 (1 034 - 912.3)	1.3±0.6
All cases with complications	11 690	7.5±5.8	7 .0 (4.0-9.0)	995.1±1 694.3	618.3 (1 014 – 1 152.3)	1.3±0.6
CKD without complication	7 258	5.7±5.5	4.0 (2.0-7.0)	780.4±1 353.8	506 (824.5 - 751.3)	-
**Total (sample hospitals)**	**18 948**	**6.8±5.7**	**6.0 (3.0-9.0)**	**912.9±1 576.1**	**574.9 (928.5 - 880.9)**	**0.8±0.7**

*LoS* length of stay, *IQR* interquartile range, *SD* standard deviation

^a^Average number of complications for those cases that had a given complication

The statistical analysis of the impact of complications revealed a considerable and highly significant difference in terms of LoS and costs (p<0.0001) between different complications of the disease (Fig. 2A-B). The complications associated with the highest number of secondary complications were hyperkalemia, volume depletion, major hypoglycemic events and fractures (Fig. 2C). The greatest number of complications were associated with the more severe cases (Stages 3-5; Supplementary tab. 3, supplementary fig 2).

Analysis of the impact of ICU costs revealed that the highest number of ICU cases were treated at university hospitals and that ICU costs are one of the key cost drivers, representing 16-26% of hospitalization costs, depending on hospital type, as shown in Table 7. Considering the whole sample, patients spent 0.2-0.4 days at the ICU. When considering only the cases that had been admitted to ICU, the averages ranged from 1.2 to 4.2 (Table 7).

**Table 7:** Length of stay and costs of intensive care in the twelve sample hospital

**Hospital type**	**Nr. of cases admitted to ICU (% of all cases by hospital type)**	**Mean LoS at ICU (days)**^**a**^	**Mean LoS at ICU (days)**^**b**^	**Median LoS at ICU (IQR)**2	**Mean cost per day at ICU (€)**	**Mean ICU cost per case (€)**	**% ICU of total costs**
University hospital	2 319 (20.3)	0.2	1.2	0 (0-0.3)	538.2	695.5	16.2
County hospital	623 (13.2)	0.4	3.0	1.3 (0.2-4)	668.5	1 984.1	26.0
City hospital	285 (10.3)	0.4	4.2	2 (0.8-5.7)	296.0	1 256.0	18.0
**Total**	**3 227 (17.0)**	**0.3**	**1.8**	**0 (0-1.4)**	**550.7**	**993.6**	**19.2**

*ICU* intensive care unit, *LoS* length of stay, *IQR* interquartile range, *SD* standard deviation

^a^calculated from all cases; ^b^calculated from cases that had been admitted to the ICU

### Epidemiology of CKD

More than 5% of all hospitalized cases in 2019 met the inclusion criteria (229 276 out of 4 150 361 discharged nationally).

**Fig. 3:**
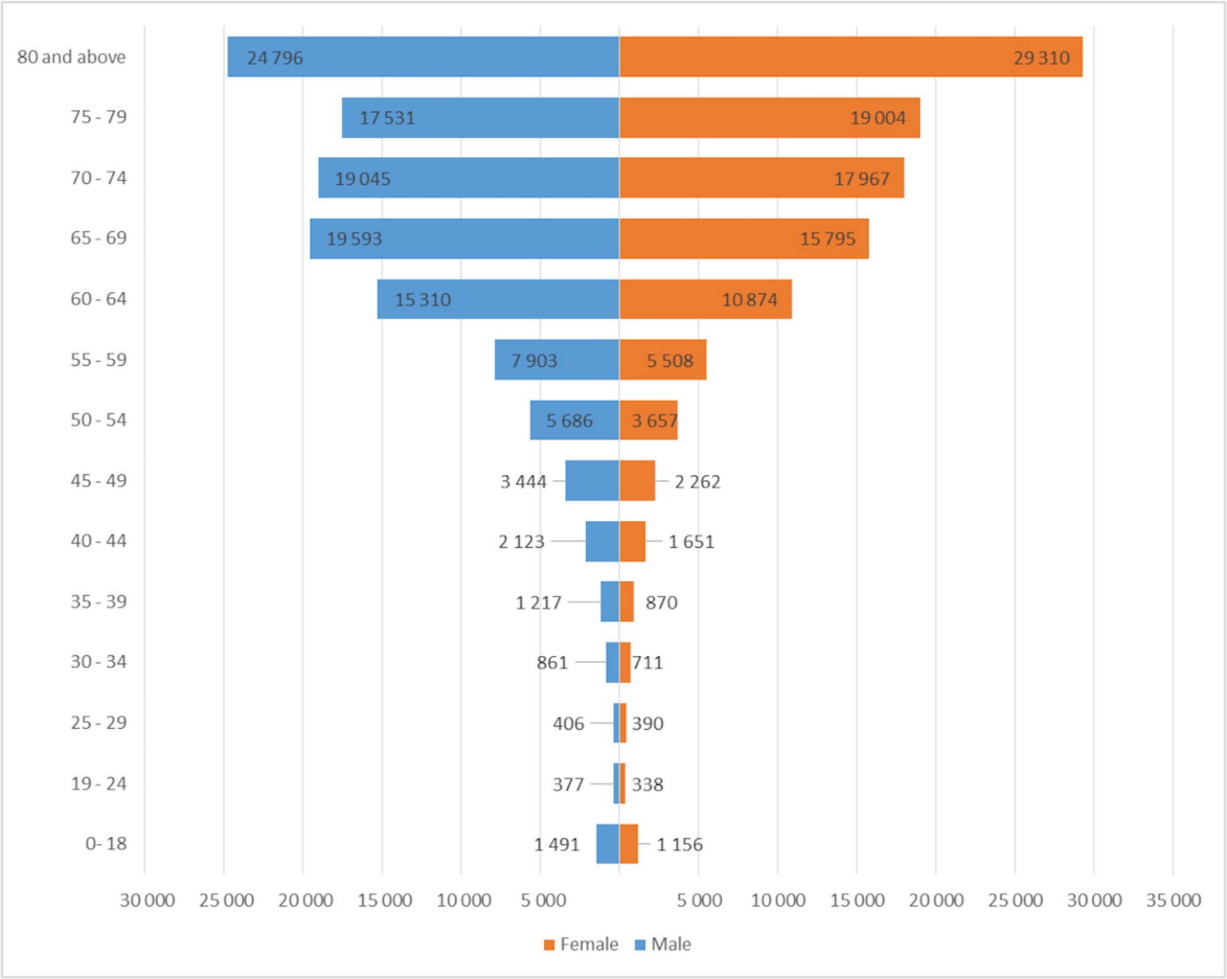
Number of chronic kidney disease cases nationally by sex and age groups

Table 8 and Figure 3 display the demographic and hospitalization characteristics of these cases. When testing for statistical significance of the differences of the age, LoS and LoS ant ICU, we found that nationally, the female patients were slightly, but significantly older and had longer LoS than males (Mann-Whitney test, *p*<0.0001). LoS at ICU was slightly higher in males, but the difference was not significant (Mann-Whitney test, *p*>0.05)

**Table 8:** Demographic characteristics and length of stay of CKD patients nationally

	**Male**	**Female**	**All cases**
	(*n*=119 783) 52%	(*n*=109 493) 48%	(*n*=229 276)
Age (years; mean ± SD)	68.3±14	70.6±13.6	69.4±13.8
LoS (days; mean ± SD)	7.3±9.6	7.5±11.1	7.4±10.4
Raw LoS at ICU (days; mean ± SD)^a^	0.4±2.6	0.4±2.2	0.4±2.4
LoS at ICU (days; mean ± SD)^b^	4.8±7.4	4.8±6.2	4.8±6.9

*LoS* length of stay, *ICU* intensive care unit, *SD* standard deviation

^a^calculated from all cases; ^b^ calculated from cases that had been admitted to the ICU

Considering the residential population of Romania in 2019, this results a general prevalence of 11.80 per 1 000. When considering the residential population above 59 years of age (4 936 655 on the 1^st^ of June) the hospitalization rate was 38.33 per 1 000 in the population aged 60 and above. As CKD is a chronic, progressive disease, the number of cases for each age group as well as the number of more severe cases (severity grades 3a-5) compared to the incipient states (severity grades 1-2) are increasing with age (Table 4). An exception to this observation might be the case of male patients above 69 years of age, since a steady decrease in case number can be observed between 70 and 79 years (Figure 3).

### Healthcare budget impact of CKD in 2019

Based on the measured average cost of PE per hospital type and the number of PEs at national level, we calculated the weighted, national average cost, resulting €917.1 per case. In 2019 there were 229 276 cases reported to the NHIH for reimbursement purposes. This means a total budget impact of €210 million.

## Discussion

Published data about the epidemiology and impact of CKD on healthcare costs in Eastern Europe are scarce. With this in mind, the present research aimed to measure the hospitalization costs generated by CKD and the one-year national-level healthcare budget impact of these costs in 2019, across Romania, regardless of severity grade. To the best of our knowledge, this study is the first that analyzes hospitalization costs related to CKD in Romania, using a *„bottom-up”* methodology. We found a national-level average cost of €917.13 per CKD case. Our epidemiological analysis resulted a number of 229 276 cases in 2019, corresponding to a one-year hospitalization rate of 11.8 per 1 000 inhabitants. This implies a total hospitalization cost budget impact of €210 million nationally. As expected, severity grades and age had a great impact on average costs per PE. The costs induced by drug administration showed a similar pattern, namely, higher drug costs were generated by the more severe cases. Regarding the differences concerning the costs between the hospitals of different complexity levels, we observed unexpectedly, that county hospitals reported the highest costs per PE (€1 032.2 vs. €900.8 and €760.1 for university and city hospitals respectively). To the best of our knowledge, there are no published data about the hospitalization costs of CKD at hospitals of different complexity levels. Nevertheless, the general clinical practice in Romania is that more severe and advanced cases with more complications tend to be transferred to more complex hospitals, thus, the university hospitals are anticipated to have the highest costs per PE. Surprisingly, the cost measurement showed that county hospitals had the highest cost per PE. The reason for this observation is unclear and raises further questions.

A recently published report by V. Jha et al., as part of the Inside CKD research program, reported CKD stage stratified cost data for 31 countries from various geographical regions, including Romania [4]. They reported a mean annual direct costs of $3 060, $3 544, $5 332 and $8 736 for stages Gr3a, 3b, 4, and 5 respectively for the 31 states and $1 737, $2 006, $2 217 and $3 353 for Romania. The reason for the difference between our results and the costs reported by Jha. et al. is of methodological nature. The detailed methodology for the cost measurement published by Jha et. al. was published by Tangri et. al in an earlier report [18]. In their report, Jha et. al extracted the one-year direct medical care-related costs, including those generated by RRTs. Another difference might be that they considered the direct cost of Gr1 and 2 to be zero. In the present article, we measured the hospitalization costs of CKD cases, regardless of severity grade.

When considering other, non-European countries, higher RRT costs have been reported by M. Nisar and collab. for Singapore, Taiwan, China, Jordan and Vietnam ($4 574, $2 901, $6 848, $16 669 and $3 489 respectively) [19]. What is more relevant for comparison with our analysis is what they reported for direct mean average non-RRT medical costs for Singapore, Japan, China, Vietnam and India ($3 412, $2 241, $4 534, $290 and $1 500 respectively). The national average that we found for CKD cases from Romania of $917.1 is close to that reported for India [19]. Nevertheless, there are several challenges in comparing reported cost data between different countries, and the interpretation of these must be cautious [20].

Our analysis of complications offers valuable insight into the economic impact of co-occurring medical conditions/diseases. These could considerably increase the costs per case, since the costs per PE were significantly higher in patients with complications (Fig. 2B). More than that, some complications are associated with considerably higher number of secondary complications (Suppl. fig.2). Several observations have to be outlined. First, the most frequent complications of CKD were heart failure and anemia (Table 6). These were associated with higher costs and longer LoS (Fig 2A-B). Secondly, the fractures appeared with low prevalence in our sample, but were associated with higher number of other complications and considerably higher costs and longer LoS (€ 1524±371.1 and 9.0±8.5 days respectively Fig. 2A-B). The causal relationship of these conditions is not clear yet. Nevertheless, these observations underscore the importance of mitigation strategies with the aim of reducing the humanistic and economic burden of CKD.

When considering the national-level costs, we first undertook an epidemiological study. This revealed that in 2019, there were 229 276 CKD cases nationally (Table 8). Considering the residential population of Romania this results a hospitalization rate of 11.8 per 1 000 inhabitants. This observation pleads for further studies in order to evaluate the existing diagnosis- and treatment gap, most importantly because the estimated prevalence of CKD is 7-13% in the general population [1, 15, 21]. In this regard, Sundström et. analyzed the epidemiology and costs of CKD across 11 countries [22]. They found, that two out of three patients that fulfilled the criteria for CKD based on laboratory results did not have a diagnostic code for CKD in the electronic health record [22]. Similar results were published by Zemplényi et. al in a recent article. They reported that only 28.6% of laboratory-confirmed cases were coded in the electronic medical record of the patients [23]. More than that, the possibility that diagnosed CKD cases were not coded in the electronic healthcare system cannot be ruled out. No such data has been published so far for Romania and it seems probable that the real number of CKD cases is higher. Nevertheless, this is in accordance with the conservative approach of our analysis. Other possible limitations with unknown impact include misspecification/misuse of diagnoses or „upcoding” (whether or not for reimbursement incentives).

Another earlier research article estimated the number of ESKD in Central and Eastern Europe [24]. In the mentioned article ESKD prevalence was 321 per one million [24]. Of these, 281 were dialyzed [24]. Additionally, there is a considerable difference between the Eastern and Western regions of Europe. Carriazzo and Ortiz compared three economically distinct regions of Europe (Western-, low-medium income Eastern Europe and high-medium income Eastern-European countries) in terms of risk factors, prevalence and outcomes of CKD [13]. They found that there is a substantial contrast in terms of disease prevalence that correlates well with the presence of some important risk factors (diabetes, raised blood pressure, obesity, physical inactivity and tobacco use) [13]. Both the risk factors and the prevalence of CKD are higher in Eastern-European countries than in Western Europeans. Nevertheless, our analysis found that out of 9 408 cases, that had the disease severity grades specified 1 772 were of severity grade 5. It is not clear how this translates into prevalence rates in the general population, but the high number of end-stage kidney disease cases (CKD grade 5 representing almost one-fifth of severity grade-specified cases) translates into high budget impact because of the RRTs.

The mean hospitalization cost in the sample hospitals was €912.92. The national, hospital complexity level-weighted mean cost was €917.13 per case. Considering the total number of cases nationally in 2019, that met the inclusion criteria, the public payer`s total inpatient expenditure was €210 273 605. This represents about 2.6% of the NHIH budget (including reimbursement for health services, drugs, medication, and national health programs, but excluding the funds for medical leave) [25]. Taking into consideration that only 1.18% of all PEs in 2019 generated this expenditure, we can consider CKD a pathology that has an important impact on the NHIH budget.

The present study has some limitations. The most important in our view is the earlier mentioned uncertainty in the proportion of miscoding (including under- and upcoding) of CKD cases in the clinical practice. Another limitation is that the cases where CKD-related disease codes were used as secondary diagnostic codes were hospitalized mainly because of other diseases, thus the hospitalization costs were generated only partially by CKD. Nevertheless, the renal condition in these cases may have contributed to the hospital costs.

ESKD as a final stage of the disease is expensive too because RRTs come into play. In our sample of twelve hospitals, out of those patients that had CKD severity stages determined, 18.8% had stage 5. In the cost measurement study reported by Jha et. al. the average haemodialysis cost across 31 countries/regions worldwide was above $57 000 [4]. The costs reported for peritoneal dialysis and the first-year costs of kidney transplant were $49 000 and $75 000 respectively [4].

It is not known how the number of ESKD (Stage 5) patients influences the costs, through the costs associated with RRTs, but it seems clear that the more advanced stages of the disease are placing considerable pressure on the healthcare budget. In our sample of twelve hospitals, the patients with stage 5 kidney disease represent 18.8% of the cases and generate 29.5% of the total inpatient costs (Table 4).

As a consequence, the importance of optimizing CKD patient care and patient-oriented research is crucial. This should aim the intervention at the earliest stage and prevent the progression to late-stage CKD and ESKD. In this regard, Sever et. al proposed a roadmap to improve the situation of kidney patients and the nephrology community in Europe [26]. This included the following steps: raising awareness about the burden and diseases/factors that lead to CKD, detection of CKD, preventing progression and minimizing complications, stimulating/prioritizing transplantation and home dialysis as RRT, dissemination/implementation of guidelines and guided therapy and encouraging the country-specific, patient-oriented research [26]. In our view, a good mitigation strategy would be a national-level screening program for the early-stage diagnosis of CKD. Within this screening protocol, the general practitioners of Romania could send their patients of at least 50 years of age once yearly for a routine kidney function assessment consisting of two routine tests (albumin-to-creatinine ratio to detect proteinuria and serum creatinine to estimate glomerular filtration rate). It is important to underscore that based on our sample the prevalence of the more advanced cases (Gr.3-5) increased after the age of 60 (Table 4.). This implies, that if these had been diagnosed ten years earlier, and the disease progression minimized, the savings would have been considerable. The measures that could contribute are: the treatment of underlying disease and managing secondary predictive factors for progression. In this regard, renin-angiotensin system blockers have a crucial role, because they attenuate the albuminuria and slow down the evolution of CKD, regardless of etiology [27].

## Conclusions

CKD inflicts high costs on the healthcare budget and indirectly upon the society as a whole. Case numbers show that CKD represents a major challenge of the public health system especially in ageing societies. Our results should warn health-policy makers about the impact of this chronic disease, and plead for the importance of mitigating strategies.

## Supplementary Information

Supplementary Material 1.

## Data Availability

The data used in this research is available on line (NHIH public national claim database). The costing data from the twelve hospitals is available for scientific purposes upon request from the corresponding author.

## Abbreviations

CKD: chronic kidney disease
ESKD: End-stage kidney disease
ICU: Intensive care unit
IQR: Interquartile range
KDIGO: Kidney Disease Improving Global Outcomes
LoS: Length of stay
NHIH: National health insurance house
PE: Patient episode
RRT: Renal replacement therapy
SD: Standard deviation
