# Supplementary material for: Epidemiology and hospitalization costs of chronic kidney disease in Romania
Source: Health Econ Rev. 2025 Apr 25;15:38. doi: 10.1186/s13561-025-00614-x (PMC12032732; doi:10.1186/s13561-025-00614-x)
Supplement: Supplementary file 1 — Supplementary Material 1. [file 13561_2025_614_MOESM1_ESM.docx]

| **ICD-10 code** | **Diagnosis code in Romanian** | **Diagnosis code in English** |
| --- | --- | --- |
| E1023 | Diabet mellitus tip 1 cu boala renala stadiu final [ESRD] | Type 1 diabetes mellitus with end stage renal disease |
| E1029 | Diabet mellitus tip 1 cu alte complicatii renale specificate | Type 1 diabetes mellitus with other specified kidney complication |
| E1123 | Diabet mellitus tip 2 cu boala renala stadiu final [ESRD] | Type 2 diabetes mellitus with end stage renal disease |
| E1129 | Diabet mellitus tip 2 cu alte complicatii renale specificate | Type 2 diabetes mellitus with other specified kidney complication |
| E1323 | Alte forme specificate de diabet mellitus cu boala renala stadiu final [ESRD] | Other specified diabetes mellitus with end stage renal disease |
| E1329 | Alte forme specificate de diabet mellitus cu alte complicatii renale specificate | Other specified diabetes mellitus with other specified kidney complication |
| E1423 | Diabet mellitus nespecificat cu boala renala stadiu final [ESRD] | Unspecified diabetes mellitus with end stage kidney disease |
| E1429 | Diabet mellitus nespecificat cu alte complicatii renale specificate | Unspecified diabetes mellitus with other specified kidney complication not elsewhere classified |
| I120 | Nefropatia hipertensiva cu insuficienta renala | Hypertensive renal disease with renal failure |
| I130 | Cardio-nefropatia hipertensiva cu insuficienta (congestiva) a inimii | Hypertensive heart and renal disease with (congestive) heart failure |
| I131 | Cardio-nefropatia hipertensiva cu insuficienta renala | Hypertensive heart and renal disease with renal failure |
| I132 | Cardio-nefropatia hipertensiva cu insuficienta cardiaca (congestiva) si renala | Hypertensive heart and renal disease with both (congestive) heart failure and renal failure |
| I151 | Hipertensiunea secundara altor afectiuni renale | Hypertension secondary to other renal disorders |
| N180 | Boala renala in stadiul final | End-stage renal disease |
| N188 | Alta insuficienta renala cronica | Other chronic renal failure |
| N1890 | Insuficienta renala cronica nespecificata | Unspecified chronic renal failure |
| N1891 | Deficienta renala cronica | Chronic renal defficiency |
| N19 | Insuficienta renala nespecificata | Unspecified kidney failure |
| Y841 | Dializa renala | Renal dialysis |
| Z992 | Dependenta de dializa renala | Dependence on renal dialysis |

**Supplementary table 1.** ICD-10 disease codes used to identify CDK cases.

| **RO-DRG v1.1 code** | **Procedure code in Romanian** | **Procedure code in English** |
| --- | --- | --- |
| K02101 | Hemodializa | Hemodialysis |
| K02203 | Dializa peritoneala continua, pe termen lung | Long- term continuous peritoneal dialysis |
| K02202 | Dializa peritoneala intermitenta, pe termen lung | Long- term intermittent peritoneal dialysis |

**Supplementary table 2.** RO-DRG v1.1 procedure codes used to identify dialysis cases.

| **Disease severity** | **Hyper K** | **Volume deple-tion** | **Major hypo-glyce-mic events** | **Frac-ture** | **Anemia** | **Hearth failure** | **All cases with compli-cations** | **No compli-cations** | **All cases** |
| --- | --- | --- | --- | --- | --- | --- | --- | --- | --- |
| Stage 1 | 5 | 1 | 3 | 1 | 23 | 77 | 110 | 293 | 403 |
| Stage 2 | 12 | 17 | 4 | 5 | 73 | 412 | 523 | 945 | 1 468 |
| Stages 1-2 | 17 | 18 | 7 | 6 | 96 | 489 | 633 | 1 238 | 1 871 |
| Stage 3a | 38 | 23 | 14 | 3 | 155 | 600 | 833 | 678 | 1 511 |
| Stage 3b | 93 | 51 | 19 | 8 | 337 | 986 | 1 494 | 694 | 2 188 |
| Stage 4 | 208 | 86 | 22 | 7 | 571 | 907 | 1 801 | 265 | 2 066 |
| Stage 5 | 234 | 52 | 12 | 7 | 933 | 489 | 1 727 | 45 | 1 772 |
| Stages 3-5 | 573 | 212 | 67 | 25 | 1 996 | 2 982 | 5 855 | 1 682 | 7 537 |
| All cases | 590 | 230 | 74 | 31 | 2 092 | 3 471 | 6 488 | 2 920 | 9 408 |

**Supplementary table 3.** The number of complications stratified by severity grades.

**Insert SUPPL FIG. 1.**

**Supplementary figure 1.** The drug costs stratified by severity grades. Significant differences are noted with *** (p<0.0005) and **** (p<0.0001). All pairs displayed highly significant differences (Stage 1 vs stage 2 p<0.0005 for all other pairs p<0.0001). The dotted line represents the median of costs of all cases that reported severity grades.

**INSERT SUPPL. FIG. 2.**

**Supplementary figure 2.** The number of complications stratified by different complications. The significant differences are shown with ** (p<0.01) and **** (p<0.0001). The dotted line represents the average number of complications calculated for all cases.
